# Development of a novel chronic kidney disease mouse model to evaluate the progression of hyperphosphatemia and associated mineral bone disease

**DOI:** 10.1038/s41598-017-02351-6

**Published:** 2017-05-22

**Authors:** Takashi Tani, Hideo Orimo, Akira Shimizu, Shuichi Tsuruoka

**Affiliations:** 1 Department of Nephrology, Graduate School of Medicine, Nippon Medical School, 1-1-5 Sendagi, Bunkyo-ku, Tokyo 113-8602 Japan; 2 Department of Metabolism and Nutrition, Graduate School of Medicine, Nippon Medical School, 1-1-5 Sendagi, Bunkyo-ku, Tokyo 113-8602 Japan; 3 Department of Analytic Human Pathology, Graduate School of Medicine, Nippon Medical School, 1-1-5 Sendagi, Bunkyo-ku, Tokyo 113-8602 Japan

## Abstract

Medial arterial calcification (MAC) and renal osteodystrophy are complications of mineral bone disease (MBD) associated with chronic kidney disease (CKD). Our aim was to develop a novel mouse model to investigate the clinical course of CKD-MBD. Eight-week-old C57BL/6 J male mice were assigned to the following groups: the control group, fed a standard chow for 6 or 12 weeks; the CKD-normal phosphorus (NP) group, fed a chow containing 0.2% adenine, with normal (0.8%) phosphorus, for 6 or 12 weeks; and the CKD-high phosphorus (HP) group, fed 6 weeks with the 0.2% adenine/0.8% phosphorus diet, followed by a chow with 1.8% phosphorus for 2 weeks, 4 weeks or 6 weeks. Serum phosphorus was significantly increased in the CKD-HP group, and associated with MAC formation; the volume of calcification increased with longer exposure to the high phosphorus feed. MAC was associated with upregulated expression of runt-related transcription factor 2, alkaline phosphatase, and osteopontin, indicative of osteoblastic trans-differentiation of vascular smooth muscle cells. A significant mineral density depletion of cortical bone was observed. We describe the feasibility of developing a model of CKD-MBD and provide findings of a direct association between elevated serum phosphorus and the formation of MAC and renal osteodystrophy.

## Introduction

The prevalence of chronic kidney disease (CKD) has increased as a function of the global aging of our society and the increasing prevalence of life-style related disease. As kidneys play an important role in the metabolism of minerals, CKD-related mineral bone disease (CKD-MBD), which includes ectopic calcification of arteries and renal osteodystrophy (ROD), is a principal complication of CKD and risk factor of CKD-related death^1, 2^. Of specific concern is the significant association between CKD and cardiovascular disease (CVD). In fact, patients with stage 5 CKD who are on dialysis have a 10–20 fold increase of CVD compared to an age- and sex-matched general population^3–6^.

Medial artery calcification (MAC), also known as Mönckeberg’s calcification, is commonly identified in patients with CKD^7–9^. MAC leads to a reduced compliance of the walls of arteries, resulting in an increase in pulse-wave velocity and systolic pressure. Over time, these altered mechanical and hemodynamic properties lead to left ventricular hypertrophy, decreased coronary perfusion, and heart failure^6, 10^. MAC of distal vessels also increases the risk of death from CVD, as well as from all-causes, in patients with non-insulin-dependent diabetes mellitus^11, 12^.

The risk for MAC-related complications increases as the stage of CKD advances^13^, with various factors associated with the formation of MAC in patients with CKD^14^. Such factors include elevated levels of serum phosphorus, calcium, parathyroid hormone (PTH), and indoxyl sulfate; and decreased levels of vasoprotective agents, pyrophosphate (PPi), fetuin A, and matrix gla-protein (MGP). Among these factors, a direct association between MAC and increased levels of serum phosphate, known as hyperphosphatemia, has been identified^13–17^. Hyperphosphatemia, which commonly develops in patients with CKD stage 4 or 5, is a strong predictor of morbidity and cardiovascular mortality in patients on dialysis^5, 6, 18–22^. In earlier stages of CKD, PTH and FGF-23 (fibroblast growth factor 23) activity is sufficient to maintain normal levels of serum phosphorus by increasing the fractional excretion of phosphorus to compensate for decreasing renal function. In addition, high levels of serum phosphate induce transformation of vascular smooth muscle cells (VSMC) into osteoblast-like cells, this transformation having been closely associated to MAC^23–25^. Trans-differentiation of VSMCs is also associated with an upregulation of runt-related transcription factor 2 (Runx2; Cbfa1), osteopontin (OPN), osteocalcin (OC), and tissue non-specific alkaline phosphatase (TNAP)^23–26^. As overexpression of TNAP on VSMCs promotes calcification^27^ and, conversely, TNAP inhibitor lowers vascular calcification (VC)^28^, TNAP is thought to play an important role in accelerating the formation of MAC. Type III sodium-dependent phosphate cotransporters, Pit-1 and Pit-2, also influence MAC formation through their modulation of intracellular uptake of phosphorus^29, 30^.

Various animal models have been developed to investigate MAC formation associated with CKD. Established techniques to induce renal failure in mice are mostly dependent on surgical intervention or transgenic manipulation. Animal models for MAC include increasing the burden of phosphorus and/or 1,25(OH)_2_D_3_ in 5/6 nephrectomy mice, the use of LDLR^−/−^, Apo E^−/−^ deficient mice fed a high-fat diet, or klotho and MGP deficient transgenic mice^31^. The use of surgical models is limited by the substantial risk of mortality and phenotypic alterations associated with procedures rather than being attributable to impairment in kidney function^31^. On the other hand, transgenic mice are often thought to provide an artificial model of CKD and MAC, with their use further limited by high cost and limited accessibility to these animals. CKD induced by adenine provides an alternative model. In this model, renal dysfunction is induced by providing animals with adenine containing feed over several weeks^32–38^. Adenine becomes a significant substrate for xanthine dehydrogenase which oxidizes adenine into 2,8-dihydroxyadenine. 2,8-dihydroxyadenine has a very low solubility and is precipitated in kidney tubules as stones, resulting in nephrolithiasis with extensive tubular dilation, necrosis, and fibrosis, accompanied by renal dysfunction^32, 33^. The adenine model has the advantage of inducing renal failure, with very little variation between animal, over a short period of time^31, 39^. In adenine-based rat CKD models, serum phosphorus elevated without dietary phosphorus load and VC developed^31, 32, 40, 41^. However, MAC does not consistently develop over time in the adenine mouse model^35^ as the C57BL/6J mouse strain, which is typically used, is quite tolerant to ectopic calcification^42^.

To overcome the limitations of current models, our aim was to develop a novel, non-surgical and non-transgenic mouse model of CKD, using an adenine-based protocol, to reproduce the clinical course of CKD-MBD.

## Results

### Between-group comparisons of physiological and biochemical parameters

**Figure 1:**
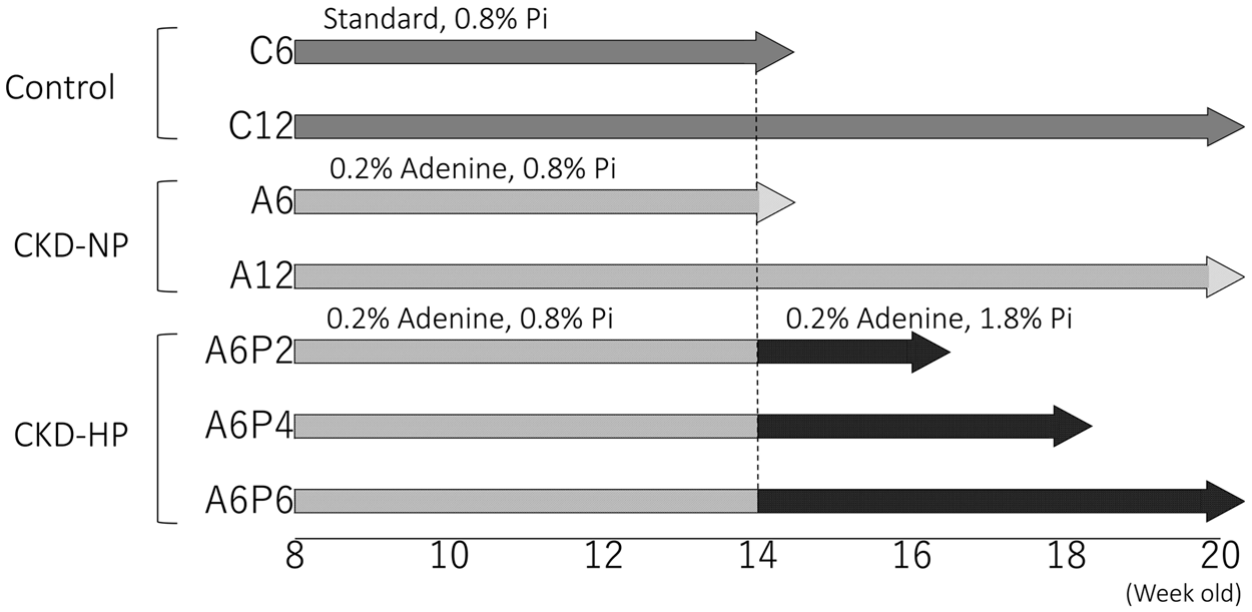
Protocols for the induction of CKD and phosphate loading. Eight-week-old C57BL/6J male mice were randomly allocated to the control, CKD-normal phosphorus (NP) and CKD- high phosphorus (HP) groups. Mice in the control groups, C6 (n = 5) and C12 (n = 5), were fed a standard chow containing 0.8% phosphorus (Pi) for 6 and 12 weeks, respectively. Mice in the CKD-NP groups, A6 (n = 5) and A12 (n = 5), were fed a chow containing 0.2% adenine and 0.8% Pi diet for 6 and 12 weeks, respectively. Mice in the CKD-HP mice were fed a chow containing 0.2% adenine and 0.8% Pi diet for 6 weeks, followed by a chow containing 0.2% adenine and 1.8% Pi 2 weeks (A6P2 group, n = 5), 4 weeks (A6P4 group, n = 5) and 6 weeks (A6P6 group, n = 5).

Eight-week-old C57BL/6J male mice were randomly allocated to the following experimental groups: control groups C6 and C12, and CKD group containing two CKD plus normal phosphorus (CKD-NP) groups (A6 and A12) and the CKD plus high phosphorus (CKD-HP) group which was subdivided into A6P2, A6P4, and A6P6 as described thoroughly in the material and methods (Fig. 1). Between-group comparisons in body weight, food intake, urine volume and serum/urine mineral metabolism parameters are summarized in Table 1. All parameters were comparable between the C6 and C12 groups. The induction phase of CKD was associated with a significant decrease in body weight among mice in the A6 group, compared to the control group. Further reduction in body weight with the high phosphorus diet in CKD-HP mice was observed. The change in body weight was not associated with a decrease in food intake (g/day) in CKD-NP and CKD-HP groups, compared to the control group. However, we identified about 4–6 fold increase in urine volume (ml/day) for the CKD groups compared to the control group.

**Table 1:** Serum, urine biochemistries and physiological parameters in CKD and control mice.

	Control		CKD				
			CKD-NP		CKD-HP		
	C6	C12	A6	A12	A6P2	A6P4	A6P6
Body Weight (g)	24.7 ± 0.6	25.8 ± 0.9	18.0 ± 0.5*	15.6 ± 0.5*^#^	16.4 ± 1.0*^#^	14.5 ± 0.4*^#^	16.0 ± 0.7*^#^
Food Intake (g/day)	2.4 ± 0.4	2.2 ± 0.6	3.8 ± 0.8*	4.3 ± 1.2	2.9 ± 1.0*	3.4 ± 1.0	3.2 ± 1.3
Urine Volume (ml/day)	0.87 ± 0.31	0.83 ± 0.36	4.16 ± 1.19*	5.19 ± 0.59*	3.44 ± 0.98*	4.01 ± 1.89*	4.00 ± 1.47*
BUN (mg/dl)	32.0 ± 6.3	32.8 ± 4.3	125.1 ± 9.5*	159.9 ± 11.2*^#^	85.2 ± 4.4*^#δ^	75.6 ± 9.8*^#δ^	70.3 ± 7.0*^#δ^
Creatinine (mg/dl)	0.12 ± 0.03	0.10 ± 0.02	0.66 ± 0.04*	0.91 ± 0.11*^#^	0.47 ± 0.06*^#δ^	0.41 ± 0.05*^#δ^	0.42 ± 0.06*^#δ^
24 h CCr (ml/min/kg)	8.49 ± 1.46	12.29 ± 3.95	1.75 ± 0.49*	1.67 ± 0.08*	1.63 ± .047*	2.45 ± 1.48*	3.20 ± 1.09*
Calcium (mg/dl)	8.08 ± 0.59	8.21 ± 0.18	8.52 ± 0.79	9.14 ± 0.80	8.66 ± 0.91	8.13 ± 0.80	8.19 ± 1.18
Phosphorous (mg/dl)	8.01 ± 2.70	7.43 ± 1.67	9.47 ± 2.14	12.3 ± 2.40*	20.9 ± 1.90*^#δ^	18.7 ± 4.55*^#δ^	18.9 ± 3.44*^#δ^
FEphos	0.10 ± 0.03	0.05 ± 0.02	0.25 ± 0.15	0.15 ± 0.04	0.71 ± 0.09*^#δ^	0.72 ± 0.10*^#δ^	0.83 ± 0.06*^#δ^
ALP (IU/min)	275 ± 40	225 ± 39	789 ± 120*	645 ± 88*	1618 ± 324*^#^	811 ± 186*	911 ± 222*
intact PTH (pg/ml)	112 ± 53	172 ± 60	1482 ± 498*	2854 ± 984*	4503 ± 689*^#^	4214 ± 820*^#^	4150 ± 813*^#^
FGF-23 (pg/ml)	104 ± 18	115 ± 12	2554 ± 892*	5415 ± 694*	4508 ± 1102*	4040 ± 951*	5666 ± 3704*

BUN, blood urea nitrogen; 24 h CCr, 24 hour creatinine clearance; FEphos, fractional excretion of phosphorus; ALP, alkaline phosphatase; PTH, parathyroid hormone; FGF-23, fibroblast growth factor 23.

The data are represented as the means ± SEM (n = 5).

*P < 0.05, **P < 0.01 vs C6 and C12 by non-repeated measures ANOVA.

^#^ *P* < 0.01, ^##^ *P* < 0.01 vs A6 by non-repeated measures ANOVA.

^δ^ *P* < 0.05, ^δδ^ *P* < 0.01 vs A12 by non-repeated measures ANOVA.

Blood levels of urea nitrogen (BUN) and serum creatinine (Cre) were significantly higher in the CKD than the control groups, with levels being lower in CKD-HP than CKD-NP mice. Increased BUN and Cre levels in CKD groups were associated with a reduced 24-h creatinine clearance (24 h CCr), compared to mice in the control group, with no difference in 24 h CCr between the CKD-NP and CKD-HP groups. Although serum phosphorus levels were comparable among A6, A12, and control mice, we identified a 1.41 to 2.78 fold increase in serum phosphorus levels among mice in the CKD-HP group relative to all other groups. Changes in serum phosphorus levels in CKD-HP mice was associated with an elevated urine fractional excretion of phosphate (%) and 24 h CCr. Serum calcium levels were comparable among groups.

A 2.35–5.88 fold increase in serum ALP activity was identified in CKD mice, compared to the control group, with the highest level identified in the A6P2 group (1618 ± 324 IU/min). CKD mice also exhibited a 13.23–40.2 fold increase in PTH, compared to the control group, with levels being higher, on average, in CKD-HP than CKD-NP mice. The level of FGF-23 was 24.5–54.4 fold higher among CKD, compared to the control group, with no difference between CKD-NP and CKD-HP mice.

### Tubular impairment and fibrotic changes in the kidneys of CKD mice

**Figure 2:**
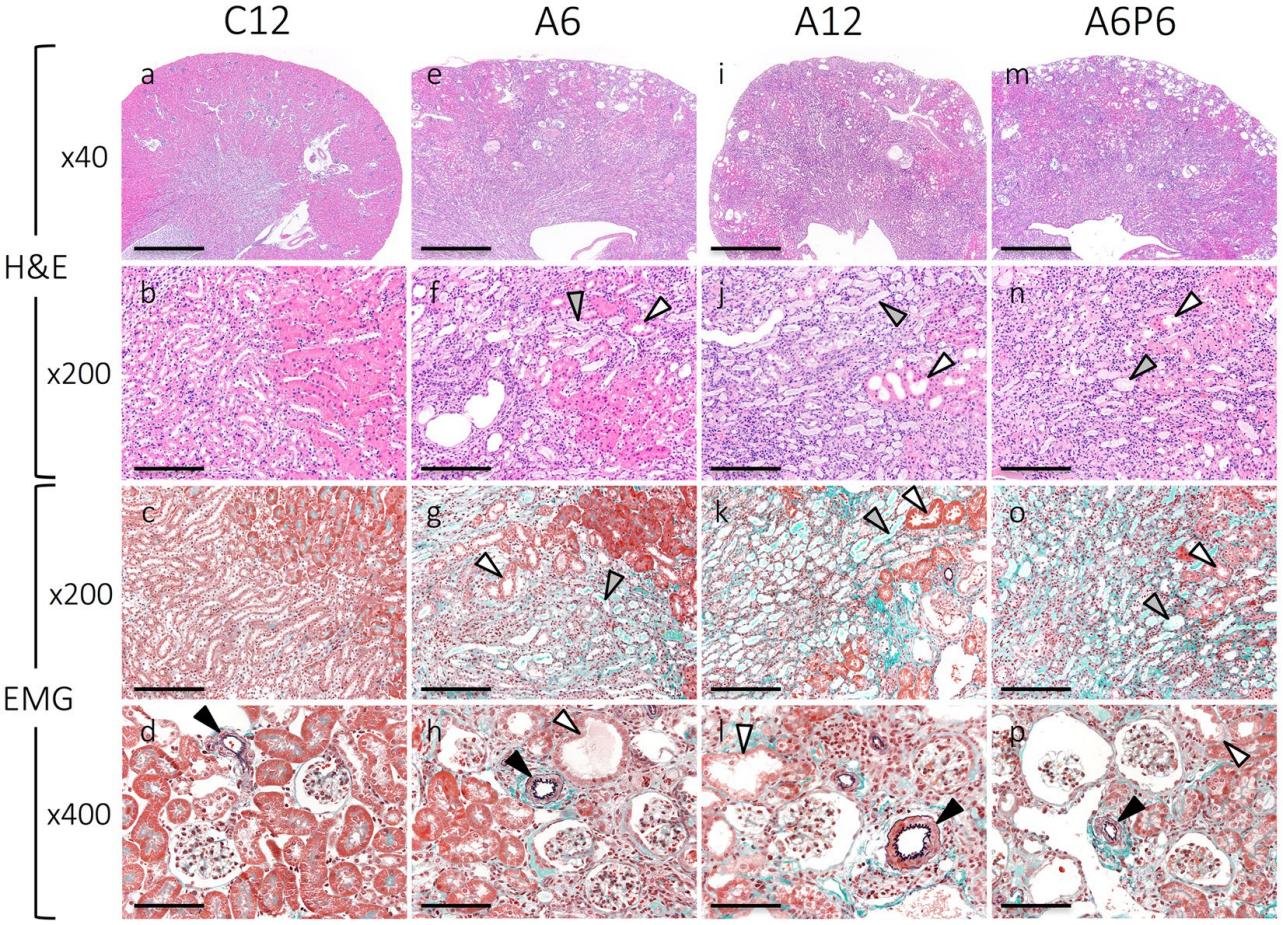
Representative histopathological findings in kidney tissues. Renal tissue was evaluated in each CKD-HP group using hematoxylin and eosin (H&E), elastica Masson-Goldner (EMG) staining. Mice from groups A6 (**e**–**h**), A12 (**i**–**l**) and A6P6 (**m**–**p**) group exhibited severe tubular impairment, with invasion of inflammatory cells, tubular luminal expansion (**f**–**h**,**j**–**l**,**n**–**p**; white arrowheads) and fibrotic changes in the stroma (**g**,**k**,**o**). Dropped tubular epithelium and casts was also evident in renal tubules (**f**,**g**,**j**,**k**,**n**,**o**; gray arrowhead). Kidney tissue of CKD mice showed atrophy, with an irregular surface (**e**,**i**,**m**). No morphological change in arterioles was identified in any group (**d**,**h**,**l**,**p**; black arrowhead). Scale bars = 500 μm for (**a**,**e**,**i**,**m**), 100 μm for (**b**,**c**,**f**,**g**,**j**,**k**,**n**,**o**), 50 μm for (**d**,**h**,**l**,**p**).

Between-group differences in the histology of kidney tissue are shown in Fig. 2. Compared to the control group, the A6 mice exhibited a higher prevalence of dropout of tubular epithelial cells, principally in distal tubules, associated with significant invasion of inflammatory cells (Fig. 2f–h) and tubular luminal expansion (Fig. 2f,g, white arrowhead). Moreover, although the glomerular structure was normal in A6 mice (Fig. 2h), the surface of the kidney appeared irregular, with evidence of atrophy (Fig. 2e). With Elastica Masson-Goldner (EMG) staining, green staining identified regions of collagen fiber hyperplasia, indicative of fibrotic changes within the kidney (Fig. 2g). These abnormal histological findings were aggravated in A12 and A6P6 mice, compared to A6 mice (Fig. 2j–l,n–p).

### Time-dependent development of MAC in the CKD-HP group

**Figure 3:**
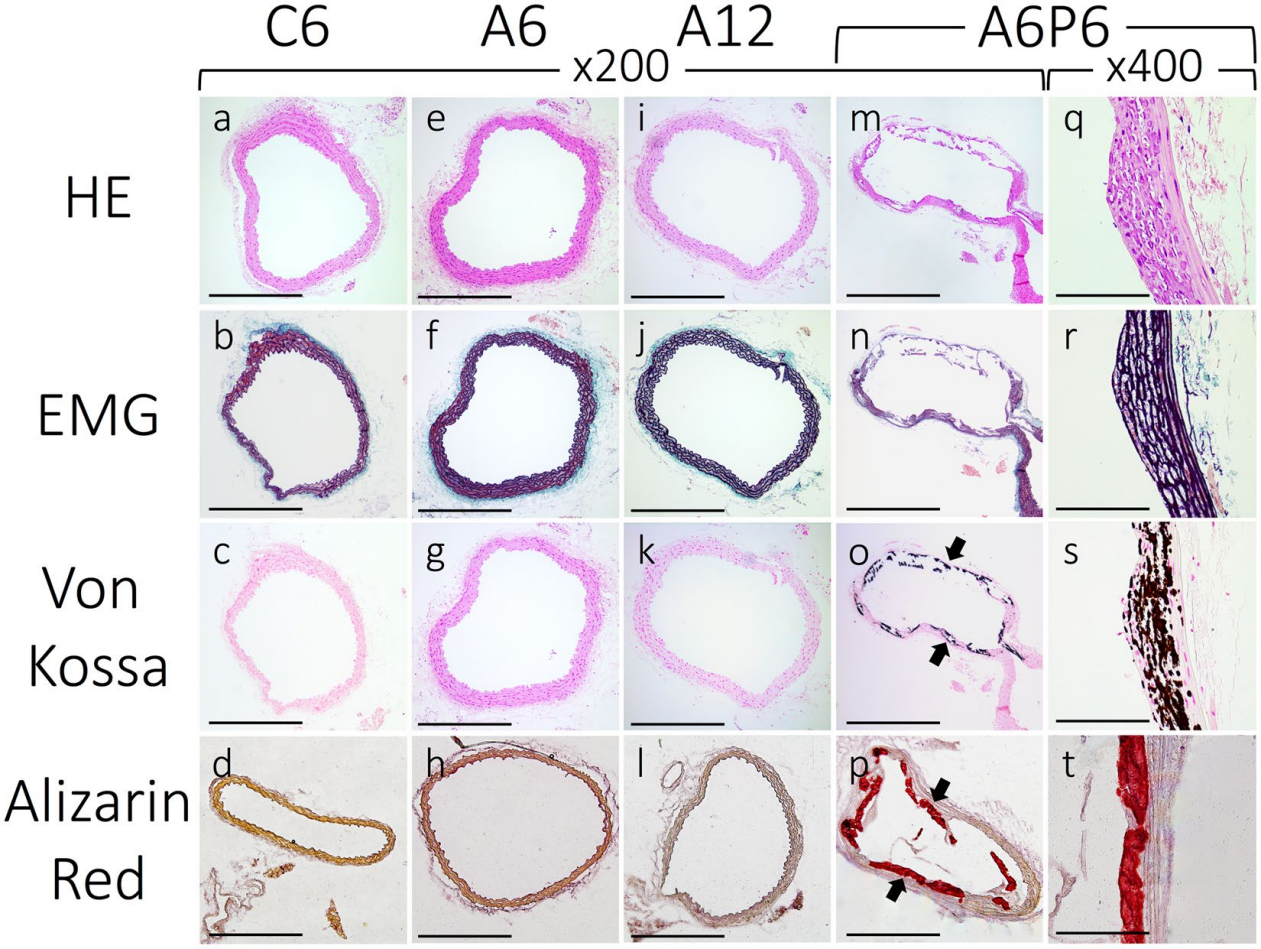
Representative micrographs of H&E, EMG, Von Kossa and Alizarin Red stained sections of the thoracic aorta. Sections of the thoracic aorta for the control (**a**–**d**), A6 (**e**–**h**), A12 (**i**–**l**), and A6P6 (**m**–**t**) groups are shown. Von Kossa and Alizarin Red staining positive legions were observed in CKD-HP mice (**o**,**p**,**s**,**t**, black arrows). Images for the A6P6 mice are shown as representatives of all CKD-HP group, as calcification in A6P2 and A6P4 groups showed similar histological features (see Supplementary Fig. S1). Scale bar, 500 μm for (**a**–**p**) and 50 μm for (**q**–**t**); H&E, hematoxylin and eosin staining; EMG, elastica Masson-Goldner staining.

**Figure 4:**
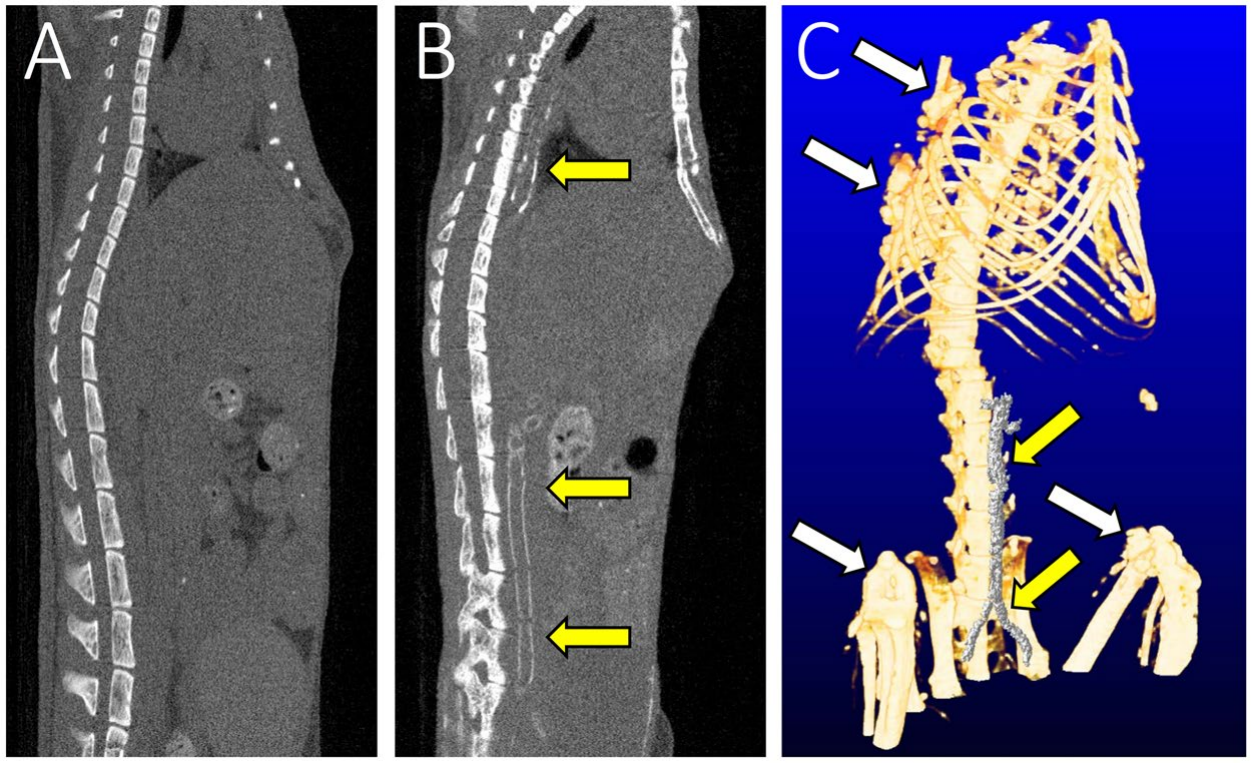
Representative computed tomography (CT) images of ectopic calcification. Positive ectopic calcifications were visible on CT images of CKD-HP (A6P6) mice in the thoracic and abdominal sections of the aorta (**B**,**C**, yellow arrows), as well as the heart, kidney and soft tissues (**C**, white arrows); no vascular calcification was observed in CKD-NP (A6) mice (**A**).

Thoracic and abdominal aorta slices, stained by hematoxylin and eosin (H&E), EMG, von-Kossa, and Alizarin Red, are shown in Fig. 3. There was no evidence of VC in the thoracic section of the aorta among mice in the control (Fig. 3c,d) and CKD-NP (Fig. 3g,h,k,l) groups. In contrast, there was clear evidence (positive von-Kossa and Alizarin Red staining) of MAC exclusively in the media, and not the adventitia, among A6P2, A6P4 and A6P6 mice, but with no evidence of cartilaginous metaplasia, inflammation or atherosclerotic lesions (Fig. 3o,p,s,t, Supplementary Fig. S1c,d,g,h). Notably, elastin layers in these calcified areas grossly appeared to be disorganized and disrupted (Fig. 3n,r, Supplementary Fig. S1b,f). Calcification of the aorta was evident on computed tomography (CT) imaging of CKD-HP mice (Fig. 4B, yellow arrows), with the aorta among mice in control and CKD-NP groups being intact (Fig. 4A). Three-dimensional (3D) reconstruction of CT images confirmed ectopic VC mainly in the abdominal aorta, up to the aortic bifurcation (Fig. 4C, yellow arrows; for 3D rotating movie, see Supplementary Video 1). Calcification of the aortic arch, heart, kidney, and of soft tissue around the rib and knee joints was also identified in CKD-HP mice (Fig. 4C, white arrows). CT imaging was performed repeatedly for the same mouse every other week between 14 and 20 weeks of age, to find VC develop severer as phosphorus loading periods get longer (Supplementary Fig. S2).

The volume of calcification in aortic tissue was quantified from CT images, confirming presence of VC only in CKD-HP groups (Table 2). Among CKD-HP groups, the volume of VC was 4.31 fold higher in A6P6 than A6P2 mice, and 1.22 fold higher in A6P6 than A6P4 mice. The same time-dependent trend was identified for the volume of VC of the heart (Table 2). The percentage calcified area (the von Kossa-positive surface) in the thoracic and abdominal aorta was calculated using Meta Morph® (version 7.10). Of all CKD-HP groups, the calcified area ratio was the lowest in A6P2 mice, increasing as phosphorus loading was prolonged in A6P4 and A6P6 mice (Table 2). This time-dependent increase in calcification among CKD-HP groups was also confirmed by *o*-Cresolphthalein Complexone (OCPC) assay of aortic tissue extracts showing clear evidence of deposited calcium in A6P4 and A6P6 mice (Table 2).

**Table 2:** Quantification of MAC by multiple analytical methods.

		Control	CKD				
			CKD-NP		CKD-HP		
		C12	A6	A12	A6P2	A6P4	A6P6
Calcified volume by qCT analysis	Aorta (mm^3^)	0.28 ± 0.07^a^	0.27 ± 0.02^a^	0.32 ± 0.13^a^	0.67 ± 0.78*^#^	2.38 ± 3.00*^#^	2.90 ± 3.98*^#^
	Heart (mm^3^)	0.35 ± 0.16^a^	0.14 ± 0.06^a^	0.21 ± 0.07^a^	0.42 ± 0.24*^#^	3.25 ± 2.78*^#^	2.81 ± 2.47*^#^
OCPC assay	Ca (umol/mg dry weight)	0.01 ± 0.00	0.01 ± 0.04	0.02 ± 0.03	0.05 ± 0.01	1.4 ± 0.1*^#δ^	5.4 ± 0.2*^#δ^
Percentage calcified surface in aorta	% Calcified surface (%)	N.D.	N.D.	N.D.	5.0 ± 10.4	11.8 ± 14.8	15.7 ± 12.8

MAC, medial arterial calcification; qCT, quantitative computed tomography; OCPC, o-cresolphthalein complexone; Ca, calcium.

The data are represented as the means ± SEM (n = 5). N.D.: not detected.

^a^Calcified volume in control and CKD-NP groups are considered to be false positive by mechanical artifacts.

*P < 0.05 vs C12, ^#^ *P* < 0.01 vs A6, ^δ^ *P* < 0.05 vs A12 by non-repeated measures ANOVA.

### Association between MAC and upregulation of mRNA levels for osteoblastic differentiation related proteins of VSMCs

**Figure 5:**
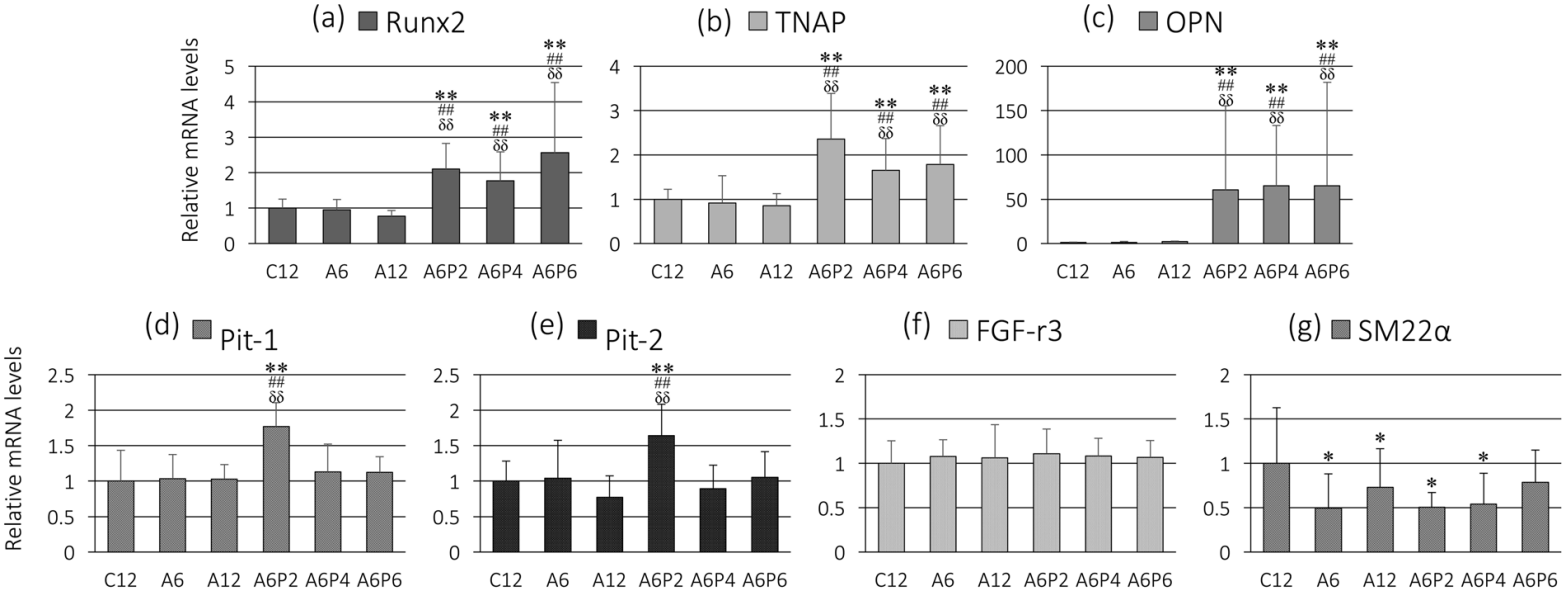
Quantitative real-time RT-PCR analysis of aortic tissue. Levels of Runx2 (**a**), tissue non-specific alkaline phosphatase (TNAP) (**b**), osteopontin (OPN) (**c**), Pit-1 (**d**), Pit-2 (**e**), fibroblast growth factor receptor 3 (FGF-r3) (**f**), and SM22-α (**g**) mRNA were determined by quantitative real-time PCR in triplicate and normalized to 18S rRNA levels. The data are represented as the mean ± SEM (n = 5); *P < 0.05 and **P < 0.01 *versus* C12; ^##^ *P* < 0.01 *versus* A6; ^δδ^ *P* < 0.01 *versus* A12; all *P*-values are evaluated by non-repeated measures ANOVA.

mRNA expression of vascular calcification-related genes was quantified by RT-PCR analysis of vascular tissue (Fig. 5). A 1.76–2.56 fold increase in Runx2 and a 1.78–2.36 fold increase in TNAP was identified in CKD-HP groups, compared to the control and CKD-NP groups (Fig. 5a,b). The upregulation in OPN expression was the most evident, with an average 60-fold increase in CKD-HP groups compared to the control and CKD-NP groups (Fig. 5c). Expression of Pit-1 and Pit-2 was only increased in the A6P2 mice (Fig. 5d,e). In contrast, expression of SM22-α, a specific marker of smooth-muscle cell, was significantly decreased in A6P2 and A6P4 group, and not in the A6P6 group (Fig. 5g). No significant change in the expression of fibroblast growth factor receptor 3 (FGF-r3), known as the receptor of FGF-23, was noted (Fig. 5f). Moreover, thresholds for α-Klotho mRNA detection were close to 40 in all groups, indicating that α-Klotho is barely expressed in aortic tissue.

### High serum phosphorus exacerbates renal osteodystrophy in CKD mice

**Figure 6:**
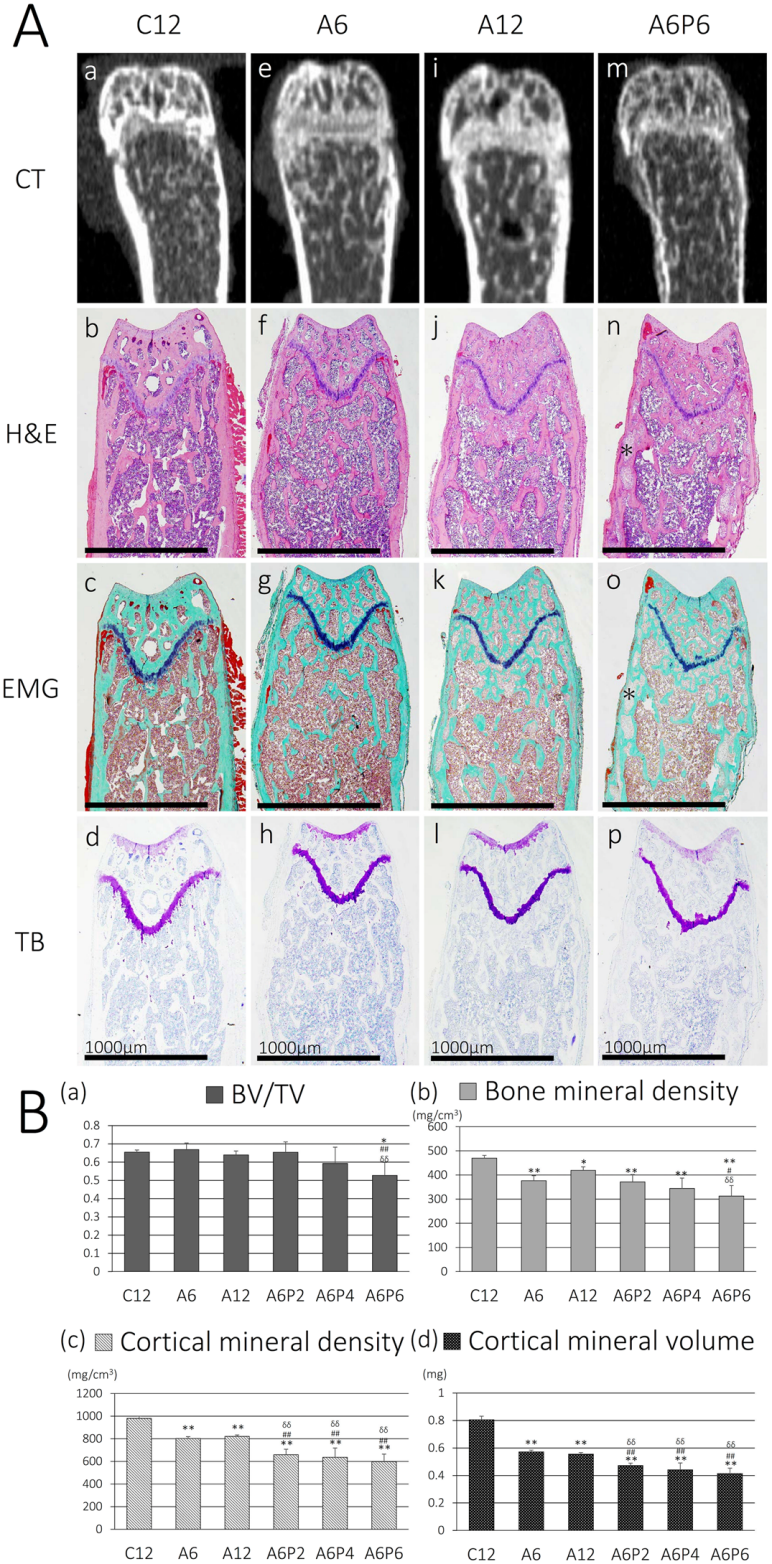
Representative histological images of the distal thighbone and morphometric parameters of distal femoral metaphysis. (**A**) Histological images from the control (C12; b–d), A6 (f–h), A12 (j–l), and A6P6 (n–p) groups are shown. For the CKD-HP groups, images from A6P6 mice are shown, with the greatest evidence of the characteristic changes in the CKD-HP groups. Although changes in the morphology of the diaphysis and irregularity of cortical bones was evident in all CKD groups (e–p), these changes were particularly strong among CKD-HP mice (m–p), with evidence of some cortical regions replaced by loose tissues (n,o; asterisk, *). Scale bar, 1000 μm. (**B**) Morphometric parameters (BV/TV, BMD, CMD, and CMV) were calculated through analysis of CT images, to identify most depressed morphometric parameters in the CKD-HP groups. CT, computed tomography; H&E, hematoxylin and eosin; EMG, elastica Masson-Goldner; TB, toluidine blue; BV/TV, bone volume/tissue volume; BMD, bone mineral density; CMD, cortical mineral density; CMV, cortical mineral volume. The data are represented as the mean ± SEM (n = 5); *P < 0.05 and **P < 0.01 *versus* C12; ^#^ *P* < 0.05 and ^##^ *P* < 0.01 *versus* A6; ^δδ^ *P* < 0.01 *versus* A12; all *P*-values are evaluated by non-repeated measures ANOVA.

Between-group differences in bone density and the structure of the femur were evaluated by CT imaging and histology (Fig. 6A). The cortex of the femur was thinned in the CKD group, particularly in CKD-HP mice, compared to the thick and homogeneous appearance of the wild-type murine cortical bone among mice in the control group (Fig. 6A–a,e,i,m). Results from histopathological imaging, performed using H&E, EMG and toluidine blue (TB) staining, were consistent with CT imaging. The cortical bone, which shows pink with H&E staining and green with EMG staining, was irregular and thinned in the CKD group compared to the control (Fig. 6A–b,c,f,g,j,k,n,o). In addition, thinning of the bone cortex was strongly observed in CKD-HP mice, especially A6P6 group, with part of the region replaced by loose tissue (Fig. 6A–n,o; asterisk, *).

The following morphometric parameters were evaluated from CT imaging of the distal femoral metaphysis: bone volume (BV), tissue volume (TV), cortical mineral density (CMD), cortical mineral volume (CMV), and bone volume fraction (BV/TV). BV/TV was significantly decreased only in the A6P6 group, compared to the control, A6 and A12 groups (Fig. 6B–a), while bone mineral density (BMD), CMD and CMV were decreased in all CKD groups, compared to the control group (Fig. 6B–b,c,d). Although CMD and CMV were lower for all CKD-HP groups (Fig. 6B–c,d), BMD was decreased only in the A6P6 group compared to the control, A6 and A12 groups (Fig. 6B–b).

## Discussion

We induced CKD using a 4–6 week diet containing 0.2% adenine as per previously published methods^33–37, 43^, with the resulting weight loss and increase in levels of BUN and Cre in the CKD mice being comparable to previously reported values^33–37^. Our results also aligned with a previous report by Jia *et al*. of an increase in intact PTH and FGF-23 rose with a prolonged administration of an adenine-containing diet^35^. Adenine-related damage to the interstitial layer of tubules, which impairs the kidney’s ability to resorb water^37^, explains the remarkable increase in urine volume in CKD mice.

The mechanism leading to lower BUN levels in CKD-HP, compared to CKD-NP, mice are unclear. There are few reports that have described improvement in kidney function by high phosphorus diet or elevated serum phosphorus, with one of these studies providing evidence of a possible mutual effect of phosphorus and adenine. Lomashvili *et al*., reported the need to provide C57BL/6J background mice with a dose of 0.45% adenine over 8 or 12 weeks to induce mild to moderate CKD at a BUN level of about 50 mg/dl when a high phosphate (2.0%(v/v)) diet load was used^44^. In contrast, it is reported that a 0.2 to 0.3% adenine loading without a concomitant phosphorus loading in C57BL/6J mice leads to severe renal failure at a BUN level of 80 to 200 mg/dl in 4 to 6 weeks, in agreement with results in CKD-NP mice^35–37^. The results of these former studies are consistent with our findings, indicative that phosphorus loading might offer a protective effect against kidney damage caused by adenine. However, as progressive renal fibrosis and exacerbation of kidney dysfunction due to phosphorus loading has previously been reported in another CKD model mice^45^, this interactive effect between adenine and phosphorus loading may be unique to the adenine-based CKD model. The specific mechanism of the adenine-phosphorus interaction remains to be clarified. On the other hand, the 24 h creatinine clearance (CCr), adjusted to body weight was comparable between the CKD-NP and HP groups, and the histological findings of kidney damage in the A6P6 mice was as severe as those found in A12 mice, with irreversible changes, such as an increase of collagen tissue in the interstitium. Therefore, we deem that all CKD groups in our study were adequate, providing a comparable CKD model with irreversible kidney damage.

The property of VC that we identified in our CKD-HP mice closely resembled the MAC formation that has previously been identified in patients on dialysis and those with diabetes, as well as in elderly individuals^46^. Our findings of an association between higher levels of serum phosphorus and intact PTH in CKD-HP than CKD-NP mice and the occurrence of VC particularly in CKD-HP mice also agrees with previous reports of a strong association between VC and phosphorus loading, both in *in vivo* and *in vitro* experiments^24–26, 47–51^. In addition, time-dependent increases in MAC of CKD-HP mice were observed on qCT imaging, histology, and OCPC assay, suggesting impacts of prolonged exposures to hyperphosphatemia on VC.

As a close relationship between phosphorus loading and VC is already known, it is important to discuss whether VC resulted from a high phosphate diet alone or CKD plus a high phosphate chow. Two studies have examined the effects of 1.5 and 2.0% high phosphorous diet in C57BL/6J background wild type mice for 8 and 12 weeks, respectively^44, 52^. In these studies, dietary phosphorus had no effect on aortic calcium content and serum phosphorus levels in these mice^44, 52^. We believe that the physiological change on wild-type animal by phosphorus loading is already established, and both CKD and phosphorus loading contributed to the formation of VC in CKD-HP mice.

In accordance with our results, elevated serum phosphorus levels were accompanied by VC in most previous CKD animal models, regardless of presence/absence of high phosphorus diets^31, 40, 41^. Moreover, in cultured cells and isolated aortic tissues, VC are formed simply by high phosphorus culture without uremic plasma^24–26^. However, there are some reports of VC by introducing CKD and phosphorus loading, without significant hyperphosphatemia using DBA/2 mice known to be susceptible to VC^49–51^. Therefore, whether hyperphosphatemia and/or high phosphate diet loading is necessary for VC in CKD models remains to be further discussed.

Gene expression in aortic tissue was consistent with our a priori hypothesis of an osteochondrocytic change in the phenotype of VSMCs associated with an upregulation in the expression of mineralization-regulating proteins^53^. Rukov *et al*. conducted RNA sequencing of VSMC-specific markers in aortic tissue in a rat model of CKD, reporting a decreased expression of SM22-α, Actin 2 and smooth muscle, associated with an increase in MGP and OPN expression^54^. Expression of Pit-1 and Pit-2, which are the only phosphate transporter present in the walls of vessels, have also been reported to be associated with VC^29, 30^. Xianwu *et al*. reported that suppression of Pit-1 inhibited VSMC calcification by phosphorus loading and was accompanied by a downregulation of Runx2 and OPN^29^. Crouthamel *et al*. reported that overexpression of Pit-2 in the Pit-1 knockdown mouse worsened VC formation, while downregulation of Pit-2 inhibited VC formation^30^. Considering their physiological role, upregulation in Pit-1 and Pit-2 expression in A6P2 mice may have promoted the inflow of phosphorus into VSMCs, leading to the osteoblastic differentiation of VSMCs. This expected mechanism of VC in our CKD-MBD model aligned well with recently proposed pathways of VC formation^17^.

The role of α-klotho expression in arterial walls and of its involvement in VC formation remains an issue of controversy. Two studies have described a plausible direct association between VC formation and decreased expression of arterial/VSMC α-klotho^55, 56^. In contrast, using real-time RT-PCR, with primers targeting different coding regions and various immune-detection techniques using several anti-α-klotho antibodies, Mencke *et al*. did not identify expression of α-klotho expression in the aorta of healthy individuals or in patients with CKD^57^, which is consistent with our results. Considering that expression of FGF-r3, a co-receptor for α-klotho, did not change, the likelihood that α-klotho is present in the walls of vessels and plays a role in resisting VC formation is moderate-to-low. This, however, does not deny the possibility that circulating α-klotho may serve as a humoral factor to protect against MAC^57^.

A high prevalence of ROD, a feature of CKD-MBD, has been reported in patients with CKD^2, 58^. While a high prevalence of VC has been clearly established in patients with CKD^7–9^, an association between VC and ROD has also been well-recognized^59, 60^. Our results are in accordance with these clinical reports. However, the reason why ROD was more severe in CKD-HP than CKD-NP mice requires further explanation. In agreement with our results, Lau *et al*. have reported a significant increase in PTH and cortical bone porosity, associated with a reduction in cortical thickness and density, in CKD mice fed a high phosphorus diet compared to CKD mice fed a diet with normal levels of phosphorus^51^. Hyperphosphatemia triggers high-turnover bone disease by an excessive secretion of PTH, which causes catabolic effects on cortical bone characteristic for hyperparathyroidism^61^. Therefore, elevated serum phosphorus may cause a distinctive reduction in cortical bone thickness and density, resulting in greater decreases of CMV and CMD levels than BMD and BV/TV in the femur of CKD-HP mice.

Our adenine-based mouse model of CKD was effective in reproducing MAC, which is a characteristic feature of CKD, without genetic manipulations, surgical interventions, or administration of drugs. Our results provided support of a strong influence of hyperphosphatemia on MAC formation, with MAC developing only in the CKD-HP group. Our findings were consistent with results from many clinical studies that have described a close association between serum phosphorus levels and VC formation and the rate of mortality among patients with CKD^5, 6, 13–20^. Furthermore, we were able to show a possible association between MAC formation and osteoblastic differentiation of VSMCs due to an increased uptake of phosphorus into VSMCs via an upregulation of Pit-1 and Pit-2 as previously reported^17, 23–25, 29, 30^.

The phenotypic changes in bones and vessels of our CKD mouse model were similar to those clinically identified in patients with advanced stages of CKD-MBD. Our objective quantification of the volume of VC, using qCT, and the calcified area ratio in histologically stained tissue provides a precise assessment of the severity of MAC. Our sequential use of CT imaging to live mammals provides a feasible method to evaluate the developing severity of MAC.

Considering that the genetic make-up of mice is more uniform than that of rats, our model is expected to be less influenced by between-animal differences that previously developed rat models of CKD. Our adenine-based protocol can also be used with other mice models, including knockout and transgenic mice. The utility of our model is significantly enhanced by its replication of CKD-related complications of VC and ROD in patients with end-stage renal disease in practice. Therefore, we expect our model will be of value to advance research in the field of CKD, including its use to elucidate the pathophysiology of CKD-related complications and to assess the effectiveness of new targeted drugs for CKD and CKD-MBD.

## Materials and Methods

### Statement of ethics

All experimental procedures were conducted in compliance with the guidelines for animal experiments at Nippon Medical School, and the study protocol was approved by the Institutional Animal Care and Use Committee of the Nippon Medical School (approval number 26–129).

### Animals

Thirty-five 8-week-old C57BL/6J mice (CLEA Japan, Inc., Tokyo, Japan) were housed in standard cages with wood chip bedding, at constant ambient temperature (21–22 °C) and humidity (40–50%), and with a 12-h light cycle. All animals had free access to tap water and their assigned diet. All mice were allowed to acclimatize to the conditions of the animal facility for a period of 7 days prior to the start of the experiments.

### Induction of CKD in the mice model

Mice were randomly assigned to the following experimental groups, with 5 animals in each group: control groups C6 and C12, two CKD-normal phosphorus groups (A6 and A12) and the CKD-high phosphorus (HP) group which was subdivided into A6P2, A6P4 and A6P6. Mice in groups C6 and C12 were fed standard pellet chow containing 0.8% phosphorus (MF; Oriental yeast Co., Tokyo, Japan) for 6 and 12 weeks, respectively (Fig. 1). Mice in groups A6 and A12 were fed a MF-based special chow containing 0.2% adenine and normal (0.8%) levels of phosphorus (Wako pure chemical industries Co., Osaka, Japan, CAS: 73-24-5) for 6 and 12 weeks, respectively. CKD-HP group mice were first fed the MF-based special chow containing 0.2% adenine and 0.8% phosphorus for 6 weeks to induce CKD. The animals were then randomly assigned to groups A6P2, A6P4 and A6P6 and fed a MF-based special chow containing 0.2% adenine and 1.8% phosphorus for a period of 2, 4 and 6 weeks, respectively. Because providing a high phosphorous diet to C57BL/6J wild type mice had no effect on aortic calcium content and serum phosphorus levels in previously published reports, we did not set such high phosphorus challenge group^44, 52^. All animals were sacrificed on the last day of their feed period.

One day prior to sacrifice, mice underwent a 24 h urine collection in individual metabolic cages, with free access to water and the assigned diet. Urine collections coincided with blood collections to provide corresponding serum data. For sacrifice, mice were anesthetized with 50 mg/kg pentobarbital, with exsanguination performed via cardiac puncture. The aorta was harvested from each animal and dissected for tissue analysis as follows: the aortic arch, fixed in RNAlater (Qiagen GmbH, Hilden, Germany) for RNA analysis; the thoracic and abdominal aorta, fixed in 10% phosphate-buffered formalin (pH 7.4; Wako pure chemical industries Co., Osaka, Japan) for histology; the abdominal aorta, snap-frozen in liquid nitrogen for calcium quantitation. The right kidney of each animal was fixed in 10% phosphate-buffered formalin (pH 7.4) for histology. The femoral bone was separated, cleaned of soft tissues, fixed in 10% phosphate-buffered formalin (pH 7.4) for 24 h and then kept in 70% ethanol solution until further use.

### Serum and urine biochemistry

Serum and urine calcium, phosphorus, creatinine, and urea levels were measured using a Hitachi 7180 auto analyzer (Hitachi high technologies Co., Tokyo, Japan). Urea concentration was evaluated using the Urease-GLDH method. Creatinine and phosphorus concentration was evaluated enzymatically, while calcium concentration was evaluated using the OCPC method. Fractional excretion of phosphate was calculated using the following formula as previously described^49^: FEphos (%) = serum creatinine × urine phosphorus/urine creatinine × serum phosphorus × 100. PTH was measured by a mouse intact PTH ELISA kit (Immutopics, San Clemente, CA, USA), and FGF-23 with an intact FGF-23 ELISA kit (KAINOS Laboratories Inc., Tokyo, Japan).

### Histology and quantitative analysis of calcified lesion

Tissue samples for histological analysis were fixed in 10% phosphate-buffered formalin (pH 7.4; Wako pure chemical industries Co., Osaka, Japan). Tissues were embedded in paraffin and H&E, EMG, von-Kossa, and Alizarin red staining performed according to standard methods. Imaging of stained sections was performed using a LEICA DM 2000 and LEICA DFC 450 C (Leica Microsystems GmbH, Wetzlar, Germany) microscope.

Identification of calcification positive areas of aortic rings was performed through analysis of tissue sections for all groups using Meta Morph® version 7.10 (Molecular Devices Co., Sunnyvale, CA, USA), with differences in tissue intensity used to differentiate calcified and non-calcified areas of the aortic ring and the ratio of calcified to non-calcified volume calculated.

### *In vivo* quantitative computed tomography (qCT) analysis

To evaluate calcified areas of the aorta, mice were anaesthetized with inhalation of 1.0 to 2.0% isoflurane (Pfizer Co., Tokyo, Japan) and subjected to CT imaging using a LaTheta LCT-200 CT scanner (Hitachi Aloka Medical Ltd., Tokyo, Japan). Quantification of aortic calcification was performed by taking slices from the aortic arch to the bifurcation of the aorta and calculating the volume of calcification using the scanner’s standard image analysis software as previously described^62^. Three dimensional (3D) image of the calcified aorta was obtained using the Amira 5 3D visualizer application (FEI Co., Hillsboro, OR, USA).

Morphometric parameters of the distal metaphysis of the femur were measured as described previously^63^, with the following parameters calculated: bone volume (BV), tissue volume (TV), bone mineral density (BMD), cortical mineral density (CMD), cortical mineral volume (CMV), and bone volume fraction (BV/TV).

### Quantification of aortic calcium

Aortic arch segments were lyophilized and decalcified with 0.6 N HCl at 37 °C for 24 h. The calcium content of the supernatant was determined by the OCPC method using a Calcium Assay Kit (Metallogenics Co., Chiba, Japan). Aortas were dried at 55 °C for 18 h, then weighed and calcium content normalized to the dry weight of the tissue (mg Ca/mg dry weight).

### Quantitative RT-PCR

Tissue RNA was extracted using chloroform (Wako pure chemical industries Co., Osaka, Japan) and Trizol (Invitrogen, Waltham, MA, USA) in a tissue homogenizer; Precellys 24 (Bertin Technologies, Saint-Quentin en Yveline, France). Tissue samples were further purified using the RNeasy Mini Kit (Qiagen GmbH, Hilden, Germany) and RNase-Free DNase set (Qiagen GmbH, Hilden, Germany). Complimentary DNA was generated with Oligo-dT primers using the ReverTra qPCR RT Kit (Toyobo Co., Osaka, Japan.) according to manufacturer’s protocol. Real-time PCR amplification was performed using the TaqMan Fast Advanced Master Mix (Life Technologies Co., Carlsbad, CA, USA) and TaqMan gene expression assay probes in a final volume of 20 μL per reaction. Proves for non-specific alkaline phosphatase (TNAP; Mm00475834_m1), runt-related transcription factor 2 (Runx2; Mm00501584_m1), Osteopontin (Mm00436767_m1), SM22-α (Mm00441661_g1), Pit-1 (Mm00489378_m1), Pit-2 (Mm00660203_m1), FGF-r3 (fibroblast growth factor receptor 3; Mm00433294_m1), α-Klotho (Mm00502002_m1) were purchased from Life Technologies Co. Amplification was carried out in an ABI 7500 Fast Real-Time PCR System (Life Technologies Co., Carlsbad, CA, USA), with one cycle at 95 °C for 20 s, followed by 50 cycles at 95 °C for 3 sec and 60 °C for 30 s. The reaction was performed in triplicate for each sample. Products were analyzed using the manufacturer’s software (SDS 1.1; Life Technologies Co., Carlsbad, CA, USA). Gene expression was normalized to the housekeeping gene 18S rRNA (Mm03928990_g1) and expressed as fold-increase using the ΔΔCt method.

### Statistical analysis

All statistical analyzes were performed using SPSS version 16.0 (IBM, Chicago, IL, USA). All values were expressed as the mean ± standard error of the mean (SEM), unless otherwise stated. Non-repeated measure analysis of variance (nrANOVA) was used to evaluate differences among the groups, with a *P*-value < 0.05 considered significant.

## Electronic supplementary material

Supplementary Figures

Supplementary Video 1
