# Supplementary material for: Development of a novel chronic kidney disease mouse model to evaluate the progression of hyperphosphatemia and associated mineral bone disease
Source: Sci Rep. 2017 May 22;7:2233. doi: 10.1038/s41598-017-02351-6 (PMC5440375; doi:10.1038/s41598-017-02351-6)
Supplement: Supplementary file 1 — Supplementary Figures [file 41598_2017_2351_MOESM1_ESM.pdf]

Development of a novel chronic kidney disease mouse model to evaluate the progression of hyperphosphatemia and associated mineral bone disease

Takashi Tani[1, 2], Hideo Orimo[2], Akira Shimizu[3], Shuichi Tsuruoka[1]

Supplementary Figures:

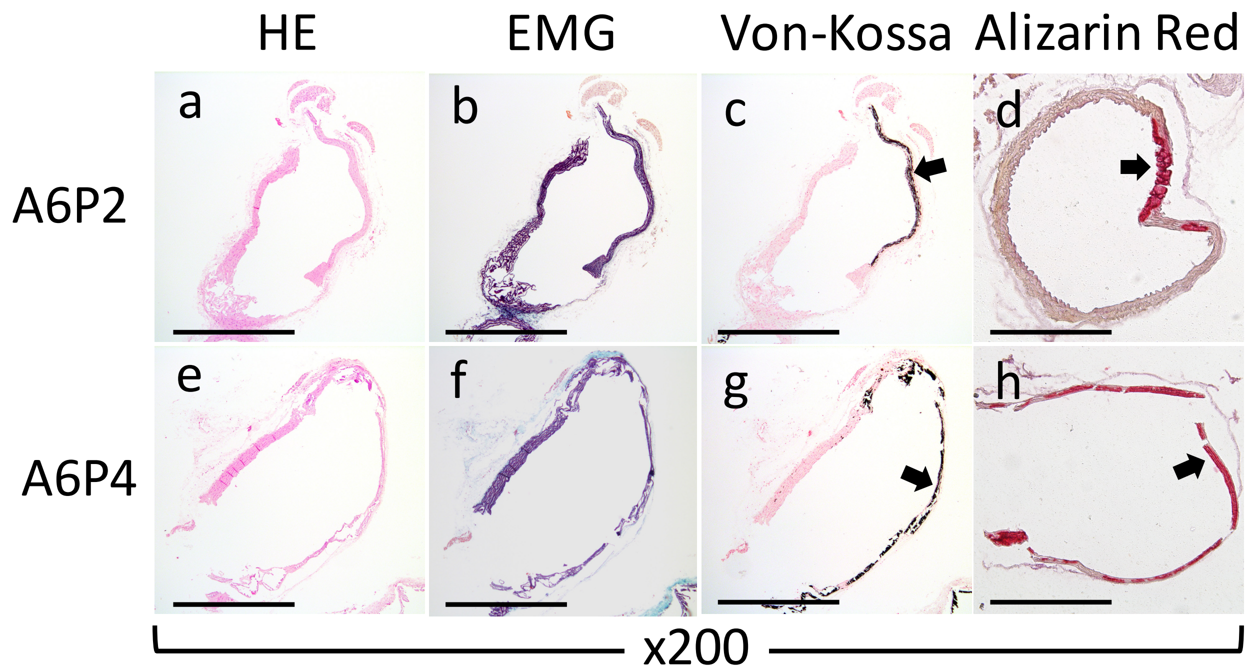

Supplementary Figure S1. Micrographs of H&E, EMG, Von kossa and Alizarin Red stained sections of the thoracic aorta from A6P2 and A6P4 mice. Sections of the thoracic aorta for A6P2 (**a-d**) and A6P4 (**e-h**) groups are shown. Medial arterial calcification was observed, as Von kossa and Alizarin Red staining positive lesion, in part of A6P2 and A6P4 groups` mice (**c,d,g,h**, black arrow). Scale bar, 500 μm; H&E, hematoxylin and eosin staining; EMG, elastica Masson-Goldner staining.

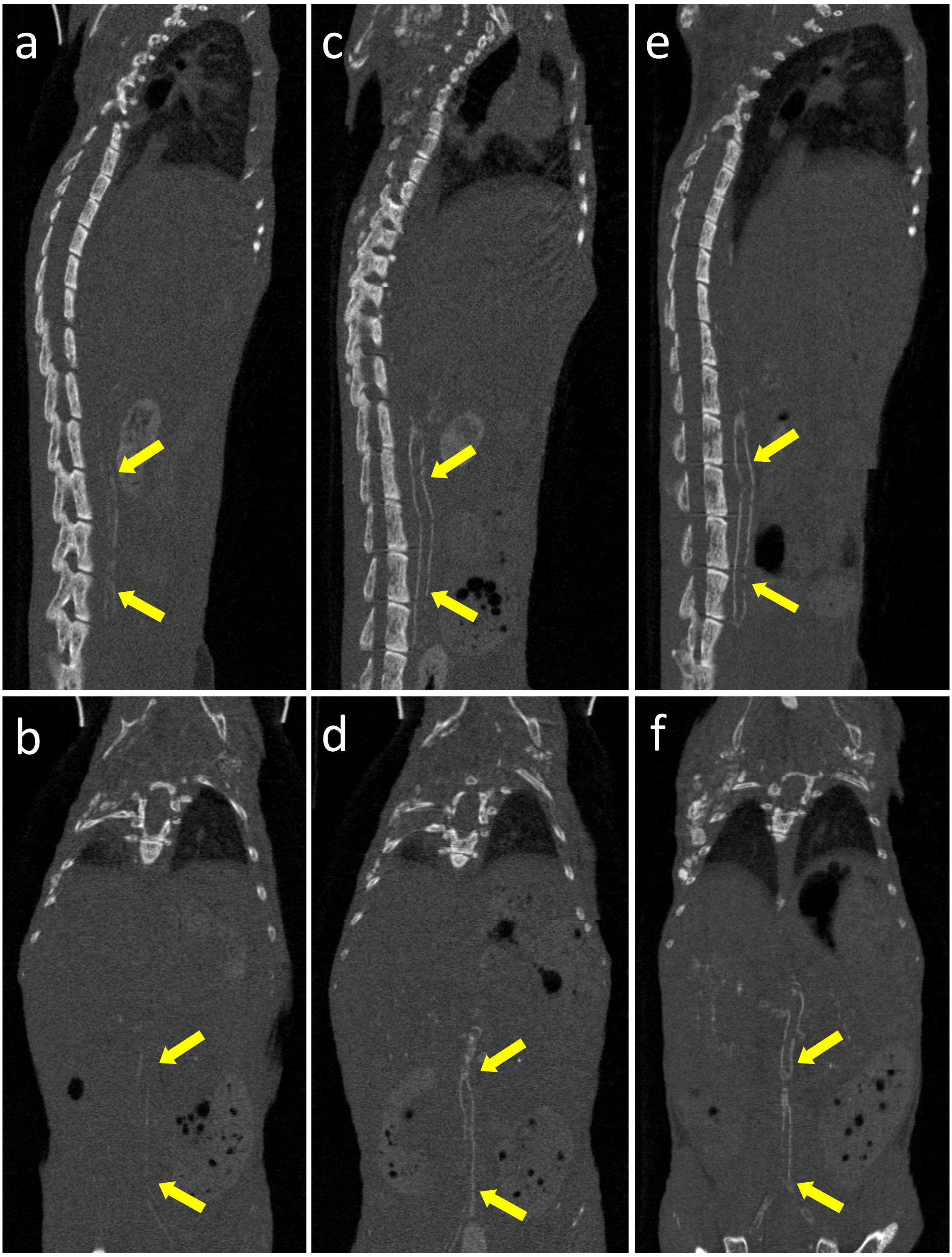

Supplementary Figure S2. Computed tomography imaging of the identical mouse between 16 and 20 weeks of age. Computed tomography (CT) imaging was performed repeatedly for identical mouse every other week between 14 and 20 weeks of age. CT images were reconstructed as sagittal plane images (**a,c,e**) and coronal plane images (**b,d,f**). Vascular calcification became prominent at 16 weeks of age, i.e. two-weeks after phosphorus loading (**a, b**, arrows). Calcification became severer as a time-dependent manner at 18 weeks of age (**c, d**, arrows) and 20 weeks of age (**e, f**, arrows**)**.
